# AI-informed conservation genomics

**DOI:** 10.1038/s41437-023-00666-x

**Published:** 2023-12-27

**Authors:** Cock van Oosterhout

**Affiliations:** 1 School of Environmental Sciences, University of East Anglia, Norwich Research Park, Norwich, NR4 7TJ UK; 2 Conservation Genetics Specialist Group, International Union for Conservation of Nature (IUCN), Gland, Switzerland

**Keywords:** Sequencing, Evolutionary genetics

Genomic data and Artificial Intelligence (AI) models will start to play an increasingly important role in conservation biology. In a recent study, Wilder et al. (2023) analysed genomic data from 240 mammal species to predict their extinction risk categories in the Red List of the International Union for Conservation of Nature (IUCN). The study processed genomic data with a machine learning model, thereby demonstrating the value of these data for the conservation of biodiversity. Wilder et al. (2023) thus show how reference genomes—and thus, genomic data more broadly—could be used for initial, cost-effective extinction risk assessments, accelerating progress made in the Red List.

## The value of genomic data in conservation

Wilder et al. (2023) found that the association between genomic data and the Red List threat category is not particularly strong. Threatened species in the Red List tend to have lower genetic diversity than non-threatened species, but the relationship is weak and variable across taxa (Brüniche-Olsen et al. 2021; Schmidt et al. 2023; Wilder et al. 2023). Similarly, genetic load and Red List category also show a very weak or inconsistent relationship (van der Valk et al. 2021; Dussex et al. 2023; Wilder et al. 2023). Thus, the relationship between the information contained in genomic data and the conservation status is unclear, particularly in recovered species (Femerling et al. 2022; Jackson et al. 2022). The question is—what is the value of genomics if these data are so poorly aligned with extinction risk as assessed by the Red List?

Genomic data are valuable precisely *because* their association with the Red List assessment is so weak. Genomic data can provide insights into aspects of extinction risk that are not reflected in the Red List. The Red List employs four criteria to assess the extinction risk of species based on a number of associated symptoms: rapid reduction in population size (criterion A); small range (area of occupancy or extent of occurrence) (criterion B); small or declining population (criterion C); very small or restricted population (criterion D), (IUCN 2012; Rodrigues et al. 2006). In addition, a very small number of species are listed based on criterion E, which relies on quantitative analysis of extinction risk (e.g., a population viability analysis using Vortex, Lacy and Pollak 2021). Clearly, there is an association between these parameters and genomic data, and conservation efforts and assessments can be enhanced using information obtained from genomic data (Paez et al. 2022; Formenti et al. 2022; Theissinger et al. 2023). A decline in population size increases the extinction risk by reducing genetic diversity, and by elevating the realised load of harmful mutations (Mathur and DeWoody 2021; Bertorelle et al. 2022). Small population size also renders species more susceptible to stochastic events and multiple Allee effects (Berec et al. 2007). However, there is a time lag between population decline and its impact on the genome, a phenomenon known as the ‘drift debt’ (Pinto et al. 2023). Nucleotide diversity is lost only slowly, and it takes many generations of drift to see this decline in genomic data (Brüniche-Olsen et al. 2021; Jackson et al. 2022; Pinto et al. 2023).

Given that the long-term effective population size (*N*_*e*_) is a function of nucleotide diversity, the *N*_*e*_ drops very slowly during population size decline as well. In turn, this raises the ratio between the *N*_*e*_ and the census population size (*N*_*c*_). Such elevated *N*_*e*_*/N*_*c*_ ratios have been reported in many threatened species (Wilder et al. 2023). Due to the slowness of genetic drift, species with *N*_*e*_ > *N*_*c*_ are set to continue to lose genetic diversity, which undermines their long-term viability. Even if the *N*_*c*_ largely recovers, such species may remain at a high risk of extinction due to continued genomic erosion and ‘drift debt’ (Jackson et al. 2022; Pinto et al. 2023). However, species that no longer meet the criteria under which they were Red Listed qualify for downlisting to a lower category of risk.

Using the number of mature individuals, the *N*_*c*_, or the increase in *N*_*c*_ (criteria A, C or D) in the assessment of extinction risk might be troublesome, especially for species that are receiving intense conservation support. Conservation efforts such as supplementary feeding are often instrumental in the demographic recovery of threatened populations (Ewen et al. 2015). However, they also relax natural selection and may help sketch an overly optimistic picture of individual fitness and population viability. In addition, in a recovering population with rapidly expanding population size, the competition between individuals relaxes, thereby reducing the efficacy of soft selection. Individuals that otherwise would have succumbed by natural selection and competition over limited resources might now be able to contribute to the gene pool of future generations. These conservation actions may thereby hide, or possibly even exacerbate, genomic erosion and its long-term threats. Consequently, a species for which conservation efforts have resulted in downlisting in the Red List may still be at risk of longer-term extinction owing to genomic erosion (Jackson et al. 2022). This can be of particular concern if the downlisting also leads to a reduction in conservation action. Conservation efforts and priorities should therefore be informed by more than simply the Red List category, as also stated in the IUCN Red List categories and criteria (IUCN 2012). In particular, decisions to reduce conservation management of downlisted species should be informed by population viability analyses that take genomic data into account.

Recently, the IUCN introduced Green Status of Species assessments as part of the Red List process, to complement the assessment of extinction risk. These assessments measure the potential of species to recover and their dependency on conservation (Grace et al. 2021). Genomic data and computer modelling approaches are exceptionally valuable in such assessments. Hence, there is now a real window of opportunity to also include genomic analyses in the IUCN’s evaluation of the recovery potential of species.

## The value of computer modelling

**Fig. 1:**
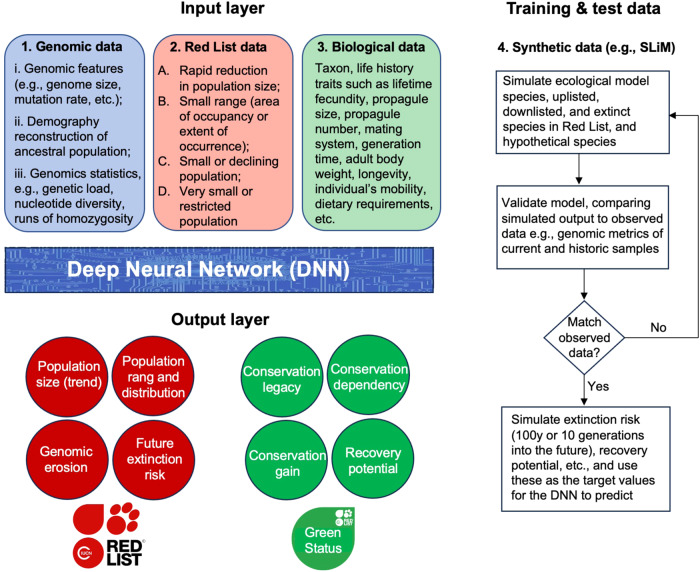
Artificial Intelligence (AI) models such as Deep Neural Networks can be trained with different data sources to predict the extinction risk and recovery potential of species. First, genomic data, Red List data, and other biological data are collected for species, including ecological model species, up- and downlisted species, extinct species, and ‘hypothetical’ species (boxes 1, 2 and 3). Next, forecasts are generated by forward-in-time computer models such as SLiM, and these synthetic data can be used as training and test data for the AI model (box 4). The SLiM model is parameterised with relevant data of the species, and its genomic data are analysed to reconstruct the ancestral demography. For ‘hypothetical’ species, a wide range of life history trait values, biological values, and demographic trajectories need to be examined. SLiM simulates the present-day (and historic) population, and these data are compared to the empirical genomic data of current samples (and historic samples, if available) to validate the model predictions. If the simulated data match the empirical data, the SLiM model can be employed to also simulate the 100 year or 10 generation forecasts. (If the match is poor, the SLiM model needs to be improved). The AI model is trained with these simulated data, using the SLiM forecasts as target values for the AI model to predict. The AI model is tested with unseen simulated data, and with empirical data of species with known conservation outcomes (e.g., extinct or recovered). Finally, once trained and tested, the AI model can assess the conservation status of species using only the genomic data, Red List data, and other biological data (boxes 1, 2 and 3). Ultimately, AI-informed conservation genomic assessments could complement the IUCN Red List and improve the Green Status assessments by providing a longer-term perspective of population viability.

Identifying the longer-term risks to population viability, e.g., over the next 100 years or 10 generations, is the real added value of genomic data (Formenti et al. 2022; Theissinger et al. 2023). But how can we use genomic statistics (e.g., nucleotide diversity) given that these metrics experience an evolutionary time-lag or ‘drift debt’ themselves? This is where computer simulations and AI models come into play. However, rather than setting the Red List category of species as the target value (as in Wilder et al. 2023), the AI model needs to be trained to predict the long-term extinction risk and recovery potential of species 100 years or 10 generations into the future (whichever is longest). These target values and training data can be generated by forward-in-time, individual-based models such as SLiM (Haller and Messer 2019) (Fig. 1). Similar to the population viability analysis carried out by the software Vortex (Lacy and Pollak 2021), SLiM can be parameterised with life history and ecological data of the focal species, and it can simulate the impacts of conservation action on population viability many generations into the future (Bertorelle et al. 2022; Dussex et al. 2021; Jackson et al. 2022; Femerling et al. 2022).

Unlike Vortex, however, this new generation of computer simulation models can also be parameterised with data of entire chromosomes (i.e., nucleotides, distribution of exons, introns and intergenic regions, the linkage map, etc.) (Haller and Messer 2019). Forward-in-time computer models can also simulate the dynamic changes in genetic diversity from the ancestral population to the present and future populations. This is insightful because that determines the size and composition of the genetic load, i.e., the proportion of masked load versus realised load (Bertorelle et al. 2022), the distributions of selection coefficients and dominance coefficients, and the frequency of harmful variants (Kyriazis et al. 2023). Furthermore, spatially explicit SLiM models can simulate the impact of habitat decline and fragmentation on genomic erosion and population viability (Pinto et al. 2023).

However, conducting such computationally intensive simulations, and collecting detailed ecological and environmental data for all >150,000 species in the Red List is simply not feasible. Furthermore, for most species we initially only possess single reference genomes. Statistics derived from a reference genome (e.g., nucleotide diversity) are more prone to the drift debt than other statistics that can only be calculated using population genomic data (e.g., the number of segregating sites or allelic richness). In addition, population genomic samples are required to infer the recent demographic trajectory (Santiago et al. 2020), which is critical when modelling the precise scenario of population decline. Hence, valuable insights can be gained first by studying a much smaller number of ecological model species for which we do possess extensive ecological and genomic data. These approaches become especially insightful if historic (museum) samples are available to study temporal trends (e.g., Dussex et al. 2021; Hogg et al. 2022; Jackson et al. 2022; Femerling et al. 2022). The empirical data of these species can then be used to simulate, hindcast, and forecast population viability to build realistic computer simulation models, and to validate their predictions (Fig. 1).

## Training and testing the AI model

Analysing a relatively small number of ecological model species might not be enough to generate sufficient training data and test data to develop an AI model. These simulations need to be expanded with extinction risk predictions of ‘hypothetical’ species that cover a wide range of parameters of all possible life histories, ecologies, and conservation scenarios. The forward-in-time SLiM simulations of these hypothetical species can help train the AI model so that it can interpolate (rather than extrapolate) from these additional simulated data (Fig. 1).

Once trained, the AI model should be further validated and tested using empirical data of species with reference genomes or resequencing data, ecological data, and known conservation outcomes. Such validation tests should also employ hindcasting to assess model predictions about species that went extinct, such as the passenger pigeon (Murray et al. 2017), mammoth (Díez-del-Molino et al. 2023), and woolly rhinoceros (Lord et al. 2020). In addition, there are dozens of species classified as extinct in the wild that possess viable captive populations (Smith et al. 2023), and these can provide important test data to validate AI model predictions. Conversely, there are hundreds of mammals and birds that have been downlisted in the Red List, some of which represent conservation success stories of species that recovered. Furthermore, the IUCN Red List documents population size trend data, and it identifies changes in extinction risk categories resulting from genuine improvement or deterioration in status, which can help test AI model predictions. If a trained AI model would be able to correctly predict the extinction or recovery of these species, it could also significantly improve the accuracy of long-term extinction risk assessments of other species with genomic data (Fig. 1).

Extinction risk assessments of such hypothetical species can also test whether the five Red List criteria might underestimate the short-term extinction risk, i.e. the risk over ten years or three generations, whichever is longer (IUCN 2012). Such simulations would help to illustrate the added value of genomic data. Forward-in-time computer models such as SLiM could simulate different conservation scenarios that threaten population viability, and these simulated populations could be assessed using criteria A to D of the Red List (IUCN 2012). The simulated populations could also be subjected to a population viability analysis using Vortex to test whether without genomic data, criterion E in the Red List is able to assess the extinction risk.

## Future challenges

A big challenge in conservation will be training AI models to assess the long-term viability of species using genomic characteristics (e.g., nucleotide diversity, genetic load, runs of homozygosity, etc.), in combination with their life history, taxonomic, ecological, environmental, and distribution data (Fig. 1). A vast amount of species-specific data are recorded in Open Access databases such as the Global Biodiversity Information Facility (https://www.gbif.org/), INSPIRE GeoPortal (https://inspire-geoportal.ec.europa.eu/), PanTHERIA (https://esapubs.org/archive/ecol/E090/184/), BirdLife International Data Zone (https://datazone.birdlife.org/), and the IUCN Red List (e.g., https://www.iucnredlist.org/resources/spatial-data-download). Unfortunately, these biodiversity data tend to be taxonomically biased (Cowie et al. 2022), which risks training and biasing AI models with incomplete data, potentially resulting in overfitting.

Integrating diverse data types with high dimensionality and sparsity is complex. Deep Neural Networks (DNN) can provide misleading predictions if the model is overfitted, something that could occur if many factors are included in the input layer as this may lead to overparametrized models. Such overfitting causes the model to only memorise the training data with limited generalisability, and solving this issue requires model simplification (Bejani and Ghatee 2021). Therefore, it is vital to address this issue during each stage of the AI model development. First, the DNN architecture is important, including parameter sharing mechanisms (e.g. convolution neural networks). Secondly, various feature engineering techniques can be utilised to reduce the complexity of the input data. For instance, dimension reduction techniques like those by Wilder et al. (2023) can facilitate joint analyses by mapping data to a lower dimensional space without significant information loss. Furthermore, feature importance ranking in Deep Learning helps to identify the most important risk factors that negatively impact population viability. This might not only help with the issue of overfitting, but it could also inform more directed conservation actions. In addition, training data need to be unbiased and span the complete range of possible variation and parameter settings. Finally, various regularisation methods, which could shrink certain model parameters towards zero (see, e.g., Goodfellow et al. 2016), should be explored during the training stage to simplify the model and increase its interpretability.

## In conclusion

The Red List assesses the extinction risk of populations and species over the next ten years or three generations, whichever is longer. As an evolutionary geneticist, I fear that the Red List is not looking far enough into the future, and that the real long-term threat posed by genomic erosion is insufficiently recognised in conservation planning (van Oosterhout 2020). AI models and genomic data are going to play an increasingly important role in conservation science, helping us to assess threats that only become visible 100 years or 10 generations into the future. If we manage to implement this new technology and data correctly, many species could be saved from extinction and assisted in their recovery, resulting in long-term viable populations. Although there are many new challenges ahead implementing AI models and genomic data, this is going to be an important and exciting research area in the next decades. DNA language models have emerged as powerful tools for processing unannotated genomic data to make molecular phenotype predictions (e.g., predicting splice sites, promoter regions, etc.) (Talukder et al. 2021). Conservation scientists will need to develop and train AI models to utilise genomic data to aid conservation. Genomic data would then not only serve as a temporary substitute for ecological data, but they would genuinely complement the Red List by providing a longer-term assessment of the extinction risk. AI models could then also enhance the IUCN’s Green Status of Species to establish the recovery potential and future conservation needs of species. AI-informed conservation genomics would constitute a genuine step change, which is critically needed given the long-term consequences of the biodiversity crisis that is challenging our planet today.
